# Skeletal muscle cell protein dysregulation highlights the pathogenesis mechanism of myopathy-associated p97/VCP R155H mutations

**DOI:** 10.3389/fneur.2023.1211635

**Published:** 2023-08-03

**Authors:** Anna Luzzi, Feng Wang, Shan Li, Michelina Iacovino, Tsui-Fen Chou

**Affiliations:** ^1^The Lundquist Institute for Biomedical Innovation at Harbor-UCLA Medical Center, Torrance, CA, United States; ^2^Department of Pediatrics, David Geffen School of Medicine at UCLA, Los Angeles, CA, United States; ^3^Division of Biology and Biological Engineering, California Institute of Technology, Pasadena, CA, United States

**Keywords:** VCP/p97, IBMPFD/ALS, iPSCs, skeletal muscle, myopathy, R155H mutation

## Abstract

p97/VCP, a hexametric member of the AAA-ATPase superfamily, has been associated with a wide range of cellular protein pathways, such as proteasomal degradation, the unfolding of polyubiquitinated proteins, and autophagosome maturation. Autosomal dominant p97/VCP mutations cause a rare hereditary multisystem disorder called IBMPFD/ALS (Inclusion Body Myopathy with Paget’s Disease and Frontotemporal Dementia/Amyotrophic Lateral Sclerosis), characterized by progressive weakness and subsequent atrophy of skeletal muscles, and impacting bones and brains, such as Parkinson’s disease, Lewy body disease, Huntington’s disease, and amyotrophic lateral ALS. Among all disease-causing mutations, Arginine 155 to Histidine (R155H/+) was reported to be the most common one, affecting over 50% of IBMPFD patients, resulting in disabling muscle weakness, which might eventually be life-threatening due to cardiac and respiratory muscle involvement. Induced pluripotent stem cells (iPSCs) offer an unlimited resource of cells to study pathology’s underlying molecular mechanism, perform drug screening, and investigate regeneration. Using R155H/+ patients’ fibroblasts, we generated IPS cells and corrected the mutation (Histidine to Arginine, H155R) to generate isogenic control cells before differentiating them into myotubes. The further proteomic analysis allowed us to identify differentially expressed proteins associated with the R155H mutation. Our results showed that R155H/+ cells were associated with dysregulated expression of several proteins involved in skeletal muscle function, cytoskeleton organization, cell signaling, intracellular organelles organization and function, cell junction, and cell adhesion. Our findings provide molecular evidence of dysfunctional protein expression in R155H/+ myotubes and offer new therapeutic targets for treating IBMPFD/ALS.

## Introduction

Inclusion Body Myopathy and Frontotemporal Dementia with early-onset Paget’s disease/Amyotrophic Lateral Sclerosis (IBMPFD/ALS) is characterized by progressive muscle weakness, bone deformities, and extensive neurodegeneration that affects muscles and bones, but also the heart and lungs due to atrophy of cardiac and respiratory muscles (1–4). Three distinct disease pathologies of variable penetrance have been identified: 1) inclusion body myopathy (IBM), an autosomal dominant myopathies with adult-onset resulting in degeneration of pelvic and shoulder girdle muscles (5, 6); 2) Paget’s disease of bone (PDB) characterized by excessive osteoblastic and osteoclastic activity, and subsequent bone remodeling with focal areas of increased bone growth, leading to bone deformities and fractures (2, 7, 8); and 3) frontotemporal dementia (FTD) affecting the frontal and anterior lobes of the brain and leading to impaired language and behavior (3, 9). This disease accounts for a substantial portion of primary degenerative dementia that occurs before age 65 (2, 7, 8). Rimmed vacuoles in IBMPFD muscle and brain tissue samples positive for p97 and ubiquitin staining is a common histological feature of the pathology. Although autosomal dominant VCP/p97 mutations have been associated with IBMPFD (9), other mutations of these genes have been linked in a wide variety of neurodegenerative disorders, including Parkinson’s disease, Lewy body disease, in both isolated familial and sporadic ALS (6), and in spinocerebellar ataxia type III (10). Specifically, VCP/p97 pathogenic mutations span the N-terminal half of the protein, which contains domains involved in ubiquitin binding and protein interactions (4). Substitution of arginine residue 155 to histidine (R155H) is the most common VCP mutation linked to IBMPFD, with mutations at this position occurring in more than 50% of IBMPFD patients (2, 3). A subset of mutations is also associated with 1–2% of amyotrophic lateral sclerosis (ALS) cases (11).

p97/VCP is a member of the AAA-ATPase superfamily and has been associated with a wide range of cellular protein pathways involved in cellular stress (12–14), Golgi and endoplasmic reticulum assembly, proteasomal and ER-associated degradation (ERAD), apoptosis (15), unfolding poly-ubiquitinated proteins (16). In particular, p97/VCP is involved in protein degradation via autophagy (16), a pathway found dysfunctional in many degenerative diseases, including myopathies (17). In IBMPDF-associated VCP/p97 mutations, abnormalities of autophagosome maturation lead to impaired autophagosome-lysosome fusion and autolysosome generation (8, 16). In addition, VCP/p97 mutations may disrupt mTOR signaling, a well-established autophagy regulator, which can contribute to IBMPFD/ALS disease pathogenesis (18).

The mouse model containing VCP/p97 mutations recapitulates the clinical manifestation of the myopathy observed in IBMPFD patients. Treatment with a VCP/p97 inhibitor leads to successful correction of the associated myopathy (1, 2).

Although a significant amount of information is available on p97/VCP mutations and the associated pathology, very little is known about differently expressed genes in this condition and how they impact the biology of the muscle. hiPSCs (human induced Pluripotent Stem Cells) derived from patients exhibiting p97/VCP mutations have widened the range of *in vitro* experiments enabling further investigation on these pathologies (19–22). To investigate the impact VCP/P97 mutations have on muscle function, we generated hiPSCs carrying the R155H mutation before differentiating them into skeletal muscle cells using established protocols (19). We then used proteomics to identify molecular mechanisms mediating VCP/P97–associated muscle dysfunction and detected dysregulated expression from several proteins involved in skeletal muscle function, intracellular organelles, and cytoskeleton organization. These findings provide an opportunity to develop new therapeutic approaches to correct the expression of disease-specific proteins.

## Materials and methods

### Human fibroblasts

Fibroblasts (GM22369, GM21752, and GM22600) were purchased from The Coriell Institute (Table 1). All diseased VCP/p97 iPSC were derived from R155H/+ patients’ own fibroblast cells and were compared to a related, unaffected control group (GM22246).

**Table 1:** Representation of groups 1–4: each Group represents a specific GM number.

Experimental group	GM	Clones	Gender	Age	Genotype	Notes
Group 1	22,369	Control 1	Male	42 years old	Wild-type	
Group 1	22,369	Control 2	Male	42 years old	Wild-type	
Group 1	22,369	Control 3	Male	42 years old	Wild-type	
Group 1	22,369	R155H/+ Clone 1	Male	42 years old	Heterozygous	
Group 1	22,369	R155H/+ Clone 2	Male	42 years old	Heterozygous	
Group 1	22,369	R155H/+ Clone 3	Male	42 years old	Heterozygous	
Group 2	22,246	Control 4	Male	40 years old	Wild-type	From unaffected family’s members
Group 2	22,246	Control 5	Male	40 years old	Wild-type	From unaffected family’s members
Group 2	22,246	Control 6	Male	40 years old	Wild-type	From unaffected family’s members
Group 2	22,246	R155H/+ Clone 4	Male	40 years old	Heterozygous	
Group 3	21,752	Control 7	Male	46 years old	Wild-type	
Group 3	21,752	R155H/+ Clone 5	Male	46 years old	Heterozygous	
Group 3	21,752	R155H/+ Clone 6	Male	46 years old	Heterozygous	
Group 4	22,600	Control 8	Female	36 years old	Wild-type	
Group 4	22,600	Control 9	Female	36 years old	Wild-type	

The table also shows the number of clones and their genotype; Control is for the isogenic cell line after CRISPR/Cas9 editing and unaffected family members of Group 2. R155H/+ is for heterozygous clones that have a mutation in one allele.

### hiPSCs generation

Human iPSCs (hiPSCs) were generated using episomal plasmids containing the following genes: Oct4 (pCE-oct3/4, Addgene #27076), Sox2 and Klf4 (pCE-hSK, Addgene #27078), c-myc and LIN28 (pCE-hUL, Addgene #27080), Dominant-negative p53 (pCE-mp53DD, Addgene #41856) and EBNA1 (pCXB-EBNA1, Addgene #41857). Nucleofection was performed using the Amaxa Human Stem Cell Nucleofector kit (Lonza, VPH 5002). Post-transduction cells were cultured in TeSR™-E7™medium (Stem Cells # 5914) for 10 days before culture in mTESR basal medium (Stem Cells #05850). Colonies were picked and expanded in mTESR basal medium in matrigel-coated plates (Corning #354277).

### Alkaline Phosphatase (AP) staining

Human iPSCs were fixed with 4% paraformaldehyde (PFA) at room temperature (RT) prior to staining using the Alkaline Phosphatase (AP) detection kit (Cell Biolabs Inc. # CBA-300) according to the manufacturer’s instructions.

### Immunofluorescence (IF) staining of hiPSCs

Human iPSCs were fixed in 4% PFA at room temperature and then stained with antibodies against the following makers: Oct3/4 (1:500 dilution Abcam #ab27985) and SSEA-4 (1:100 dilution Abcam# ab16287) and AlexFluor-488 conjugated antibodies against human TRA-1-60 (1:100 dilution BD Pharmigen #560173) in 0.1% Triton (Fisher X-100) and and 1% Fetal Bovine Serum (FBS) in PBS. For secondary antibodies goat IgG (1:250 in PBS, Invitrogen #A11055) and mouse IgG (1:250 in PBS, Invitrogen #A11055) were used to detect Oct3/4 and anti-SSEA-4, respectively. Cell nuclei were counterstained with Hoechst 33342 (10 ng/mL of Thermo Scientific #H1399).

### Gene editing of hiPSCs R155H/+ using RNA-based methods

gRNA and DNA templates were purchased from Integrated DNA technology (IDT) as follows: Guide RNA 5′-CCACAGCACG CATCCCACCA-3′), H155R-Reverse Complement 5′-ATCTGTTT CCACCACTTT GAACTCCACAGCACGCATGCCACCACGTACA AGAAAAATGTCTCCTGCGAGAGCAAACAGTA-3′), (R155H- Reverse Complement 5′-ATCTGTTTCCA CCACTTTGAACTC CACAGCACGCATGCCACCA TGTACAAGAAAAATGTCTCC TGCGAGAGCAAACAGTA-3′. Briefly, a pre-annealed mixture of Atl^®^ CRISPR-Cas9 crRNA Guide 1, tracrRNA ATTO™550 [(200 μM each, IDT #1075928), and Atl^®^ S. p. HiFi Cas 9 Nuclease V3(IDT, cat #1081061] were prepared following manufacturer instructions. The Ribonucleoprotein (RNP) complex was prepared by mixing 240 pmol of Atl′ CRISPR-Cas9 crRNA Guide 1 + CRISPR-Cas9 tracr RNA ATTO^™^550, 208 pmol of Alt-R^®^ Cas9 enzyme, and 240 pmol of 100 μM Ultramer DNA Oligo (Integrated DNA Technology). Before nucleofection, 10.8 μM of Alt-R^®^ Cas9 Electroporator Enhancer was added to the nucleofection mixture and integrated into cells. The emission of red light indicates the introduction of the CRISPR/Cas9 complex into the cells by the tracrRNA-ATTO and the yield of the transfection. The cells were let to grow for a few days and then dissociated into single cells for clonal selection.

### Digestion with Sph1 restriction enzyme and sequencing clones

iPSC DNA (~310 bp) was amplified by PCR (Platinum SuperFi PCR Master Mix, Invitrogen #12358) using custom Reverse primers (IDT). Primers (VCP-Ex5-F 5′-TGGAGTTGGGGAGAGGTAGGG-3′, and VCP-Ex5-R 5′-AAAATCGGATACTGGAATCAGGGAGA-3′). PCR product was digested with Sph1 HF (New England Biolabs #R3182L), and clones positive for the Sph1 digestion were purified using agarose gel (Qiagen, #28704). Before sequencing, amplicons were treated with Exonuclease I (10 U Thermo Fisher Scientific # EN0581) and FastAP™ Thermosensitive AP (1 U, Thermo Fisher Scientific #EF0654). The samples were mixed and incubated at 37°C for 15 min, followed by incubation at 85°C for 15 min. Samples were sent for sequencing and analyzed using the software FinchTV (Geospiza, Inc., WA, United States) (23).

### Lentiviruses and packaging plasmids production

The doxycycline (Dox)-inducible PAX7 system consisted of two lentiviral vectors: the rtTA-FUGW lentivirus that carries the reverse tetracycline transactivator and hPAX7-pSAM2 that carries the tetracycline response element (TRE-promoter) to control hPAX7 induction and ires-GFP (19). Viruses were generated by cotransfection with packaging plasmids in 293 T cells (24). The virus supernatant was collected at 24- and 48 h post-transfection and concentrated by centrifugation (22,000 g for 2 h). iPSCs were transduced using 13 MOI for pSAM2-PAX7 (calculated on 293 T cells) using spin infection (centrifugation at 2,600 g for 1.5 h and 4 h recovery). A transduction rate greater than 20% GPF-positive cells was used in the downstream experiments.

### Skeletal muscle differentiation

For skeletal muscle differentiation we used previously published method (20). Briefly, embryoid bodies (EBs) were generated in mTESR and then grown using EB differentiation medium (IMDM (1X) + GlutaMAX^™^-I, Gibco #31980-030) supplemented with 15% FBS (Atlanta #S11150), 10% Horse Serum (Gibco #26050-088), 4.5 mM Monothyoglycerol (Alfa Aesar), 25 mg of Ascorbic Acid (Acros, Organics), 100 mg of human Holo-Transferrin (RD System #2914-HT), and 1% Penicillin/Streptomycin. To perform myoblasts differentiation, we induced Pax7 expression using 0.75 mg/mL Doxycycline for 4 days (Millipore Sigma # D9891) in EB medium. After 7 days of differentiation, EBs were plated as a monolayer culture, and we isolated GFP and PAX7-positive myoblasts using FACS sorting (BD FACS Aria III). Cells were then expanded and terminally differentiated into myotubes using the myotube differentiation medium: DMEM low glucose (Gibco #11885–084), supplemented with 20% of KnockOut SR (Gibco, #10828–028), 10 mM of SB431545 (Cayman Chemical #13031), 10 m of DAPT (Adipogen), and 1% Penicillin/Streptomycin.

### FACS analysis

To evaluate the efficiency of myoblast formation, we stained the expanded myoblasts with α-Alpha 7 integrin-PE (AbLab, Cat #67–0010-05) and α-human CD29-APC (eBioscience #17–029942) as previously described (25).

### Immunostaining for myosin heavy chain (MHC)

Myotubes were fixed in PFA 4%, permeabilized with 0.3% Triton X-100 in PBS for 20 min at room temperature and stained using a primary antibody against MF-20 (1:20, Developmental Studies Hybridoma Bank – DSHB) and an Alexa-Fluor 555-tagged secondary antibody anti-mouse (1:250, Invitrogen #A28180). Nuclei were stained with Hoechst, and imaging was performed using Evos FL Fluorescence microscope.

### qRT-PCR analysis of skeletal muscle cells

Total RNA was extracted using the kit Direct-zol^™^ RNA Miniprep Plus (Zymo Research #R2072) following the manufacturer’s protocol. The cDNA was synthesized using 1 μg of the total RNA with the reverse transcriptase (Bioline, Sensi FAST Kit). Gene expression levels were measured by RT-PCR using the cDNA with the Sensi-Fast Hi-Rox Kit (Bioline #Bio-82,020). The target genes’ relative expression was normalized to that of glyceraldehyde 3-phosphate dehydrogenase (GAPDH Hs02786624-g1 20x Applied Biosystem). The expression of the following genes was analyzed PAX3 (Hs00240950), Myf5 (Hs00929416-g1), MyoD1(Hs02330075-g1), and Myogenin (Hs01072232-m1, Applied Biosystems).

### Statistical analysis

Data were expressed as mean +/− SEM, and statistical significance was measured using the unpaired Student’s *t*-test. The statistical significance was set at *p* ≤ 0.05.

### Western Blot analysis

Protein samples and dual plus molecular weight ladders were separated by SDS-PAGE using Precast Gels with a 4–15% gradient (Bio-Rad #4561083). Proteins were transferred to nitrocellulose membranes (Bio-Rad #170–4,159) using the Bio-Rad Trans-Blot Turbo Transfer System for 7 min. Total proteins on membranes were detected using the Ponceau S staining. Membranes were blocked with 5% non-fat milk in TBS-T and incubated with primary antibodies against human Myf5 (Abcam #125301), and MyoD1 (Abcam #16148) in TBS-T with 2.5% non-fat milk at 4°C overnight. HRP-conjugated secondary antibodies (1:3000), anti-rabbit-HRP (Invitrogen #31460), and anti-mouse HRP (Invitrogen #31430) were used, were incubated with the membrane in TBST with 2.5% non-fat dry milk for 1 h at room temperature. Membranes were exposed to the chemiluminescence reagent (Millipore #WBKLS0500) for 2 min at room temperature and visualized using Chemidoc (Bio-rad).

### Mass spectrometry

Myotubes from each Group were lysed using a lysis buffer (10 M Urea, 40 mM HEPES pH 7.5, 200 mM NaCl) containing a Protease & Phosphatase Inhibitor Cocktail (Fisher #78440) and 10 mM MG132. Proteins were digested in MS buffer (0.1 M Tris–HCl, Boston BioProducts #BT-P-920) containing 0.5 M TCEP (Fisher #20491, prepared in MS buffer), 0.5 M 2-chloroacetamide (MP Biomedicals #ICN15495580), 0.25 μg/μL Lys-C (FujiFilm Wako Chemicals United States Corporation #125–05061, prepared in MS grade water), incubated at 37°C for 4 h with shaking at 750 rpm; 100 mM CaCl_2_ and 0.5 μg/μL Trypsin (Fisher #90058) were added to the samples and incubated at 37°C for 20 h with shaking at 750 rpm. The digested samples were then desalted using C18 columns (Fisher #89870) and dried using a vacuum centrifuge. Before running mass spec samples, samples were dissolved in 0.2% FA solution, and peptide concentration was tested through Pierce Quantitative Fluorometric Peptide Assay (Fisher #23290). LC–MS/MS experiments were performed using an EASY-nLC 1,000 connected to a Q Exactive Orbitrap Mass Spectrometers (Fisher). 0.25 μg sample was loaded onto an Easy Spray Column (25 cm x 75 μm, 2 μm C18, ES802, Fisher) and separated over 195 min at a flow rate of 0.5 μL/min with the following gradient: 2–35% B (180 min), 35–85% B (5 min), and 85% B (10 min). Solvent A consisted of 99.9% H_2_O and 0.1% formic acid, and solvent B consisted of 19.9% H2O, 80% ACN, and 0.1% formic acid. A full MS scan was acquired at 70,000 resolutions with a scan range of 350–2000 m/z, the AGC target was 1 × 106, and the maximum injection time was 100 ms. MS2 scan was acquired at 17,500 resolutions with a scan range of 200–2000 m/z, the AGC target was 5 × 104, the maximum injection time was 64 ms, and the isolation window was 2.0 m/z. System control and data collection were performed by Xcalibur software.

Proteomic data processing was performed through Proteome Discoverer 1.4 (Fisher) using the Uniprot human database and the Sequest HT Search Engine. The search allowed for a precursor mass tolerance of 10 ppm, a minimum peptide length of 6, and a minimum peptide sequence number of 1. Upon identification of dysregulated protein expression levels from the control sample and correction for false discovery rate (t-test <0.05), we analyzed interaction protein using the STRING program.

## Results

### Generation of hiPSCs and isogenic control lines

**Figure 1:**
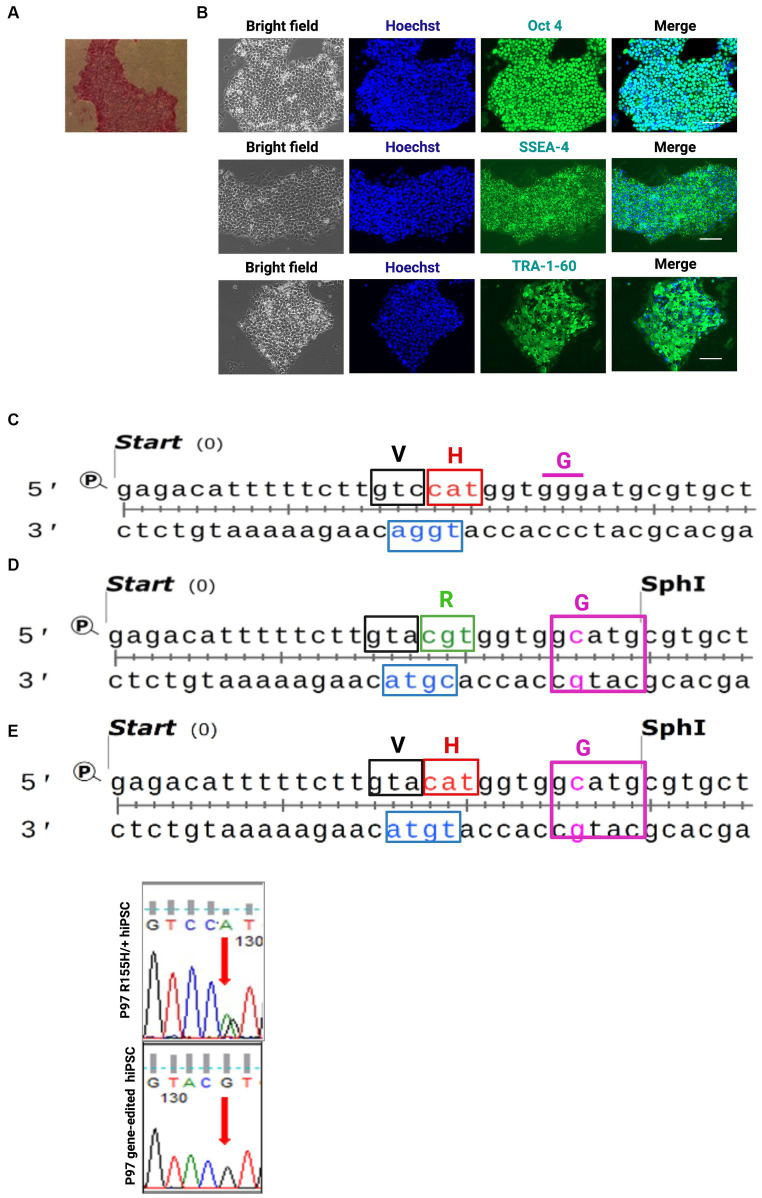
Human iPSC (hiPSCs) derived from patients’ fibroblast harboring R155H mutation in the p97/VCP gene and the isogenic control cells. **(A)** hiPSCs colony for alkaline phosphatase. **(B)** Pluripotency was confirmed via immunofluorescence. Bright-field nuclei stained with Hoechst (Blue), Oct4; SSEA-4 and TRA-1-60 (green), Scale Barr 400 nm. **(C)** sequence of patient iPSC exon 5 carrying the p97/VCP^R155H/+^. **(D)** sequence of the DNA template used to generate isotype control and to correct R155H mutation. **(E)** sequence of the DNA template used to generate R155H from control cells. Red codon R155H, green codon H155R, Cas9 PAM blue. H histidine, R arginine, V valine, G glycine. Histogram of sequenced Exon 5 showing G base at the place of the A base.

We generated hiPSCs from three human fibroblasts harboring the p97/VCP ^R155H +/−^ mutation and from one unaffected related control. Successfully reprogrammed hiPSCs were validated with Alkaline Phosphatase assay and the SSEA-4, TRA-1-60, and Oct4 markers, confirming their pluripotency (Figures 1A,B and Supplementary Figures S1A,B). All clones derived from a specific patient were labeled as belonging to the same Group. Selected hiPSC p97/VCP^R155H+/−^ clones were corrected to p97/VCP^isoWT^ using the CRISPR/Cas9 and homology recombination (HR) as previously described (26–28). The mutation R155H/+ is in the exon 5 of the VCP/p97 gene (Figure 1C). We used a homologous recombination DNA template containing a Guanidine (G) in codon CGT (coding for Arginine) to replace the Adenine (A) in codon CAT. The DNA template also had a missense mutation for Glycine to introduce the Sph1 digestion site and a missense mutation for Valine, V (GTA) to disrupt the PAM sequence (Figure 1D). To generate p97/VCP R155H from isogenic Control, we prepared a similar DNA template containing the CAT codon to replace histidine in the arginine 155 (Figure 1E). Successful modifications were verified through sequencing (Figure 1F).

### Differentiation of hiPSCs into myotubes skeletal muscle cells

**Figure 2:**
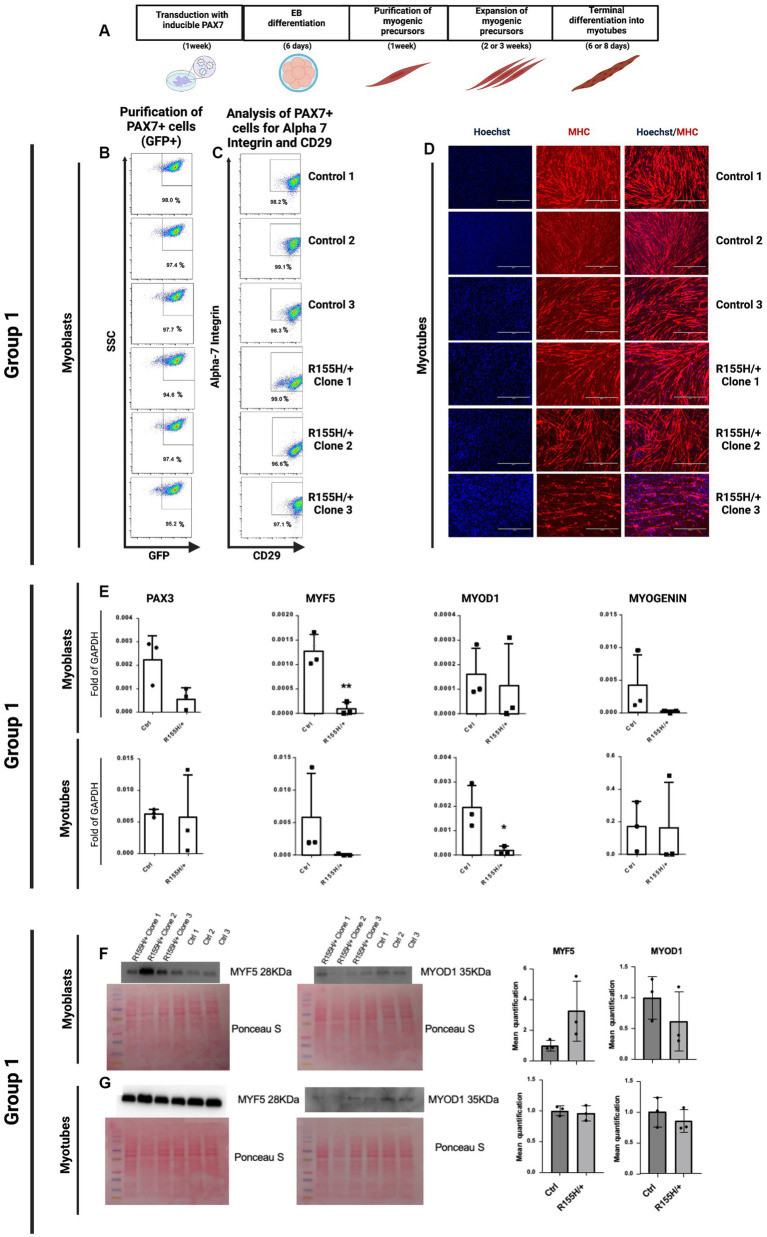
Differentiation of hiPSC into skeletal muscle fibers via expressing PAX7. **(A)** Schematic representation of skeletal muscle differentiation protocol and terminal differentiation of skeletal muscle cells into myotubes. **(B,C)** Representative FACS profile of PAX7-induced proliferating myogenic progenitors. Group 1 myoblasts were previously purified by FACS selection of only GFP-positive (PAX7^+^) cells and then expanded. **(C)** After 1 week of expansion, the myogenic precursors were stained for two early skeletal muscle markers, Alpha 7 integrin and CD29. The percentage indicates cells staining positive for GFP, Alpha 7 Integrin, and CD29. SSC side scatter. **(D)** Myogenic progenitors were differentiated into myotubes over 6–8 days, and immunofluorescence determined the myotube formation. Blue nuclei as stained with Hoechst. Red, Myosin Heavy Chain (MHC), myotubes; Scale Barr 400 nm. **(E)** qRT-PCR of Group 1 myoblast and myotubes, showing expression of PAX3, MYF5 (Marker of myogenic precursors: myoblasts), MYOD1, and MYOGENIN (Late marker of terminally differentiated skeletal muscle cells). Western Blot analysis for the expression analysis and quantification of the proteins Myf5 (28KDa) and MyoD1 (35KDa) in myoblasts **(F)** and myotubes **(G)**, where are showed the cropped blot images **(F,G)**. Their corresponding uncropped full-length blot images are also represented (Supplementary Figures S5A,B).

hiPSC clones from Control and R155H/+ groups (for a total number of 8 lines) were differentiated into skeletal muscle following a multi-step schematic protocol that included transduction of the hiPSCs with an inducible PAX7 expression, Embryoid Bodies (EBs) formation, purification, and expansion of myogenic precursors, and finally the formation of the multinucleated muscle fibers or myotubes (Figure 2A) (20). Diseased and isogenic control iPSCs were successfully differentiated into myoblast expressing PAX7 (GFP+ cells), CD29, and alpha-7 integrin (Figures 2B,C). Further differentiation was performed to generate multinucleated myofibers expressing skeletal muscle marker MHC (Figure 2D). The same procedure was applied to the cells of all other iPSC groups 2–4 (Supplementary Figures S2A–I).

Next, we measured gene expression of the myoblast’s progenitor markers PAX3 and MYF5 and of differentiated myoblasts MYOD1 and MYOGENIN in both Control vs. diseased myoblast. Our results reveal that p97/VCP^R155H +/−^ myoblasts had lower expression of PAX3 and MYF5 than controls and that p97/VCP^R155H +/−^ myotubes had a significative lower expression of MYOD1 (Figure 2E). We obtained similar results with all 4 groups (Supplementary Figure S2J). Using Western Blot analysis, we assessed MYF5 and MYOD1 protein levels in myoblasts and myotubes in Group 1 cells. We detected higher levels of MYF5 protein in the p97/VCP^R155H +/−^ lines, but it did not reach statistical differences. MYOD remained unchanged (Figures 2F,G). The proteins MYF5 (28KDa) and MYOD1 (35KDa) are shown in cropped blot images (Figures 2F,G), and their corresponding full-length blot images are shown as well (Supplementary Figures S5A,B).

### Global proteomic analysis in skeletal muscle fibers of R155H/+ and isogenic control

**Figure 3:**
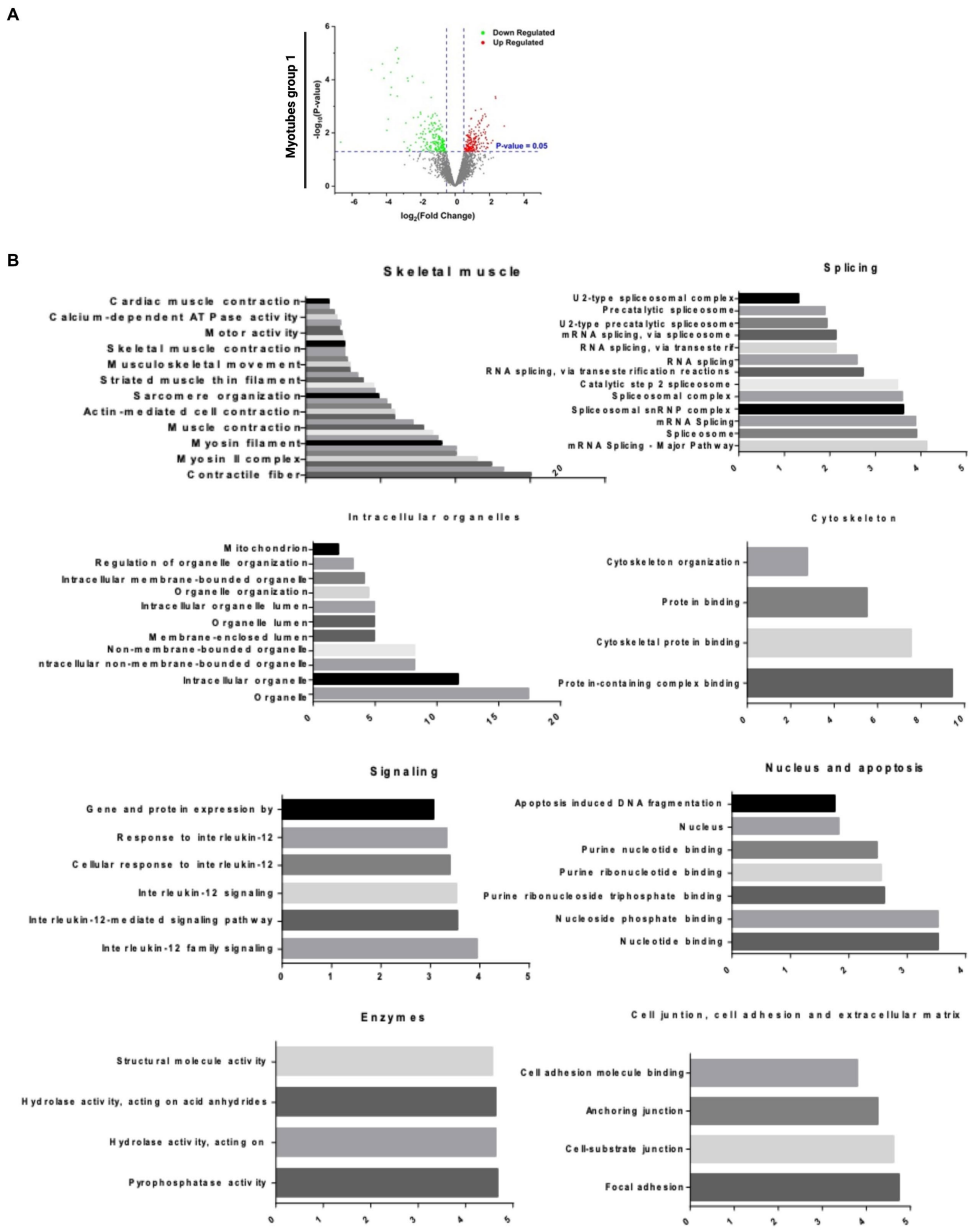
Bioinformatics analysis of proteomic results. **(A)** Volcano plot analysis of statistically significant myotubes (*p*-value >0.05). The p-value is represented in the Y ax, and the fold change in the X ax. The green dots are the down-regulated proteins, and the red dots are the upregulated proteins. **(B)** DE Pathways analysis of Group 1 myotubes: skeletal muscle, splicing, intracellular organelles, cytoskeleton, signaling, nucleus and apoptosis, enzymes and cell junction, cell adhesion, and extracellular matrix. DE Pathways analysis of all myotubes: skeletal muscle, splicing, intracellular organelles, cytoskeleton, signaling/cancer and enzymes, and nucleoside binding.

To investigate the global differences in IBMPFD/ALS myotubes, we performed an unbiased proteomic analysis in group 1. Proteomic analysis revealed dysregulated protein (*p* value < 0.05) shown in the Volcano plot. Red dots represent upregulated proteins, and the green dots are down-regulated proteins (Figure 3A). Similar pattern of dysregulated myotubes is present in groups 1–4 (Supplementary Figure S3A). Pathway analysis showed that many dysregulated proteins are involved in skeletal muscle, intracellular organelles, cytoskeleton organization, cellular communication, and signaling. Each pathway comprises serial sub-pathways that specify the protein functions (Figure 3B and Supplementary Figure S3B).

### R155H/+ impacts skeletal muscle, autophagy, and mitochondrial function

**Figure 4:**
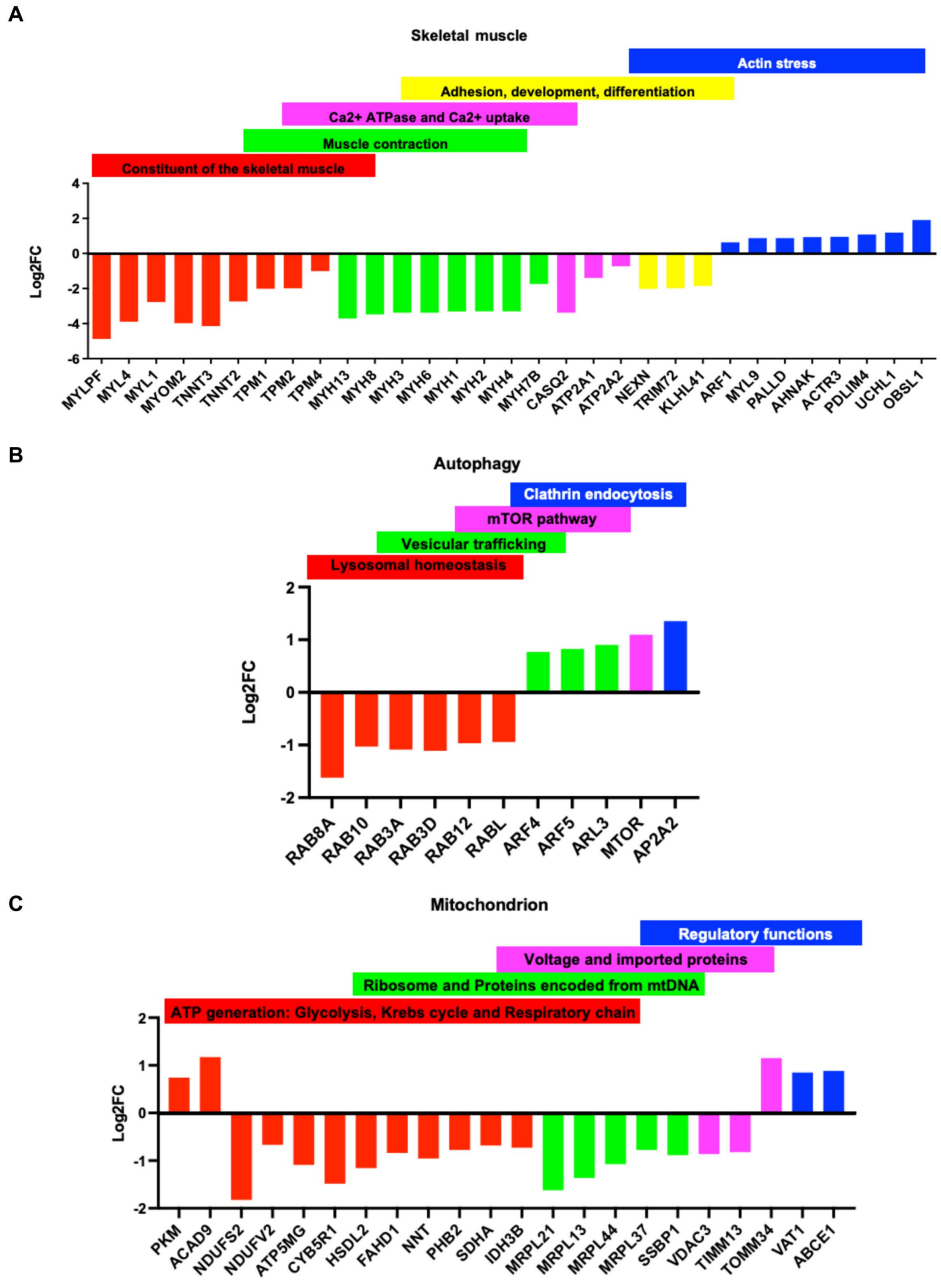
Dys-regulation of proteins in the Group 1 myotubes: Classification of dysregulated proteins (*p*-value<0.05 cut off) based on fold changes (logFC) in the Group 1 myotubes. Y ax logFC: negative values, downregulated proteins positive values, upregulated protein. X ax: name of the proteins. **(A)** constituent of the skeletal muscle (red), muscle contraction (green), Calcium ATPase and uptake (purple), adhesion, development, and differentiation(yellow), and actin stress (blue). **(B)** Dysregulated proteins in the autophagy: lysosomal homeostasis (red), vesicular trafficking (green), mTOR pathway (purple), and clathrin endocytosis (blue). **(C)** Dysregulated proteins in the mitochondria: ATP generation: glycolysis, Krebs cycle and respiratory chain (red), mitochondrial ribosome and protein encoded by the mitochondrial DNA, mtDNA (green), voltage and Import proteins (purple), regulatory functions (blue).

The myopathy described in IBMPFD/ALS leads to muscle weakness (1, 2). We found that several constituents of the skeletal muscle architecture, including myosin, troponins, and tropomyosin, as well as several involved in muscle contraction, were downregulated in p97/VCP^R155H +/−^. Other proteins involved in muscle filament sliding, sarcomere organization, myosin complex, and phosphatase activity were downregulated. In addition, several proteins required in the reuptake of cytosolic calcium into the sarcoplasmic reticulum and in calcium-binding function were downregulated. In contrast, proteins involved in actin stress, a mechanism of myosin and actin contraction in non-muscle fibers, were upregulated (Figure 4A). It was also proposed that the actin stress function as the template for sarcomere formation in cardiac cells, suggesting that diseased cells may not fully differentiate into mature sarcomere (29, 30). A similar protein expression pattern was found in the myotubes of groups 1–4 (Supplementary Figure S4A).

p97/VCP is crucial for autophagosome maturation (4, 8), and multiple studies report that VCP/p97 mutants impair autophagy mechanisms (9, 16). We found several proteins involved in lysosomal homeostasis and endosome recycling that were downregulated in diseased myotubes. On the contrary, proteins involved in vesicular trafficking and clathrin endocytosis were upregulated (Figure 4B). Our data show that mutation of VCP/p97 in IBMPFD disrupts mTOR signaling, a serine/threonine kinase that contributes to myopathy and which has been showing to worsen the severity of the disease (18) (Figure 4B). Proteins with very similar biological functions were also found in the myotubes groups 1–4 (Supplementary Figure S4B).

Dysfunction in mitochondria, unique organelles essential for various cellular processes such as energy metabolism, calcium homeostasis, lipid biosynthesis, and apoptosis, is a known prevalent feature of many neurodegenerative diseases and motor neuron disorders such as ALS. Disruption of mitochondria structure, dynamics, bioenergetics, and calcium buffering has been extensively reported in ALS patients (31). In diseased myotubes, we found that several proteins involved in the formation of mitochondrial respiratory complexes were downregulated. In contrast, proteins involved in glycolysis and assembly of complex 1 were upregulated, suggesting an enhancement of glycolysis mechanisms as compensation for dysfunctional mitochondria. In addition, proteins relevant for mitochondrial translation, DNA inheritance, and protein import were downregulated, indicating dysfunctional mitochondria (Figure 4C and Supplementary Figure S4C). Finally, proteomic data of diseased myotubes revealed that proteins involved in cellular stress responses, such as DNA repair, protein degradation, and protein folding, were dysregulated (Supplementary Figure S4D) (12, 14, 32). p97/VCP also has a chaperone function, and its mutations interfere with cellular methylation, affecting numerous protein features such as turnover, activity, and molecular interactions (13). Some dysregulated proteins promote p53/TP53 degradation and protein degradation (Supplementary Figure S4D).

## Discussion

p97/VCP is essential in many cellular functions, including proteasomal degradation and autophagosome maturation. In addition, this protein complex is required to dislocate proteins from the endoplasmic reticulum (ER) to the cytosol during the endoplasmic reticulum-associated degradation (ERAD) (15).

The most common mutation of VCP/p97 in IBMPFD patients is located on the R155H site (3, 9). IBMPFD affects the function of muscles, bones, lungs, and the brain. Pathological features in IBMPFD samples include rimmed vacuoles found in p97 and ubiquitin-positive muscle tissues and nuclear inclusions in p97 and ubiquitin-positive neurons in brain tissues (6). IBMPFD mice exhibiting the R155H and A232E mutations showed progressive weakness and atrophy of skeletal muscles (2). To understand the impact of VCP/p97 mutants on myotubes and myoblasts, we generated patients-derived iPSC and differentiated them into myoblasts and myotubes. We found that while diseased and Control cells could generate myotubes, diseased myotubes had a decreased expression of MyoD1. Although MYF5 protein levels were increased in diseased myoblasts, no significant difference in MYOD and MYF5 protein levels were found, probably due to high variation among clones.

Our global protein analysis on myotubes revealed that several proteins involved in key muscle function structure, contraction, and calcium uptake were downregulated, suggesting a dysfunction in muscle contraction ability in p97/VCP^R155H +/−^ cells. We also discovered that diseased muscle had increased protein levels involved in actin stress. These contracting proteins are usually expressed in non-muscle fibers, such as in smooth muscle cells, and function as a template to generate mature sarcomeres (29, 30), suggesting an abnormal contraction mechanism in this pathology. Our findings agree with previously published data providing molecular evidence of myopathy.

p97/VCP mutants inhibit proper autophagy, a degradation system that processes proteins too large for the proteasome. Our proteomic data suggest that proteins involved in protein degradation via proteasome or lysosome are downregulated. MTOR, a key negative regulator of autophagy initiation, is upregulated in our study. It was previously shown that mTOR activity is inhibited in R155H mutant myoblasts, which promotes autophagosome formation, and inhibits autophagosome maturation, thus blocking the mTOR function downstream of the autophagy pathway (18). In addition, an increase in mTOR inhibition was shown to worsen the myopathy associated with the disease, suggesting that the accumulation of autophagosomes that cannot proceed to full maturation is more harmful than impaired autophagy. Therefore, increasing mTOR may counteract the mTOR inhibition (18) by decreasing autophagosomes that cannot progress to maturation due to VCP/p97 mutation (4, 8).

VCP/p97 mutation dysregulates several mitochondrial proteins. Mitochondria dysfunction is a prevalent feature of many neurodegenerative diseases and motor neuron disorders such as ALS. Disruption of mitochondria structure, dynamics, bioenergetics, and calcium buffering has been extensively reported in ALS patients (31). Skeletal muscle requires a lot of energy, and abundant mitochondria provide the energy in physiological conditions. Patients harboring VCP/p97 mutation have mitochondria dysfunction, resulting in reduced ATP synthesis and dysregulation in the mitochondria function (33). Our proteomic analysis reveals a decrease in the expression of proteins involved in ATP formation and the import of proteins into the mitochondria, providing molecular targets responsible for mitochondrial dysfunction.

## Conclusion

Our data show how VCP/p97 mutations can impair several essential biological processes in skeletal muscles, such as autophagy and mitochondria function, leading to disease progression in IBMPFD/ALS patients. Identifying the protein for which the expression is dysregulated in this disease shines a light on key therapeutic targets for developing a treatment that can reduce the severity of the disease and slow down its progression.

## Data availability statement

The original contributions presented in the study are publicly available. This data can be found in PRIDE under the accession number PXD044004: https://www.ebi.ac.uk/pride/archive/projects/PXD044004.

## Author contributions

AL, MI, and T-FC wrote the main manuscript. AL prepared Figures 1–4, Supplementary Figures S1–S5, and Table 1. FW and SL analyzed Figures 3, 4 and the Supplementary Figures S3, S4. All authors contributed to the article and approved the submitted version.

## Funding

This work was supported by funds from the National Institute of Neurological Disorders and Stroke (NINDS), R01NS102279.

## Conflict of interest

The authors declare that the research was conducted in the absence of any commercial or financial relationships that could be construed as a potential conflict of interest.

## Publisher’s note

All claims expressed in this article are solely those of the authors and do not necessarily represent those of their affiliated organizations, or those of the publisher, the editors and the reviewers. Any product that may be evaluated in this article, or claim that may be made by its manufacturer, is not guaranteed or endorsed by the publisher.

## Supplementary material

SUPPLEMENTARY FIGURE S1 Human iPSCs (hiPSCs) fibroblast-derived from patients harboring R155H mutation in the VCP/p97 gene and their screening selection of clones edited by the CRISPR/RNA method. iPSC diseased of groups 2-4 stained for **(A)** alkaline phosphatase. **(B)** pluripotency markers, Bright field, nuclei stained with Hoechst (Blue), Oct4; SSEA-4 and TRA-1-60 (green); Scale Barr 400nm.

SUPPLEMENTARY FIGURE S2 Differentiation of R155H/+ and WT p97 hiPSC-Derived myogenic progenitors into muscle tissue following PAX7 induction. Group 2 **(A-C)**, group 3 **(D-F)**, and group 4 **(G-I)** myoblast were previously purified by FACS selection of only GFP positive (PAX7+) cells and then expanded Representative FACS profile of PAX7 induced proliferating myogenic progenitors **(A, D, G)**. After 1 week of expansion, the myogenic precursors were stained for two early skeletal muscle markers, Alpha 7 integrin, and CD29. The percentage indicates cells staining positive for GFP, Alpha 7 Integrin, and CD29. SSC side scatters **(B, E, H)**. Myogenic progenitors were differentiated into myotubes over 6-8 days, and immunofluorescence determined the myotube formation. Blue nuclei as stained with Hoechst. Red, Myosin Heavy Chain (MHC), myotubes; Scale Barr 400nm.

SUPPLEMENTARY FIGURE S3 Bioinformatics analysis of proteomic results. **(A)** Volcano plot analysis of statistically significant myotubes (P-value >0.05) in groups 1-4 myotubes. The P-value is represented in the Y ax, and the fold change in the X ax. The green dots are the down-regulated proteins, and the red dots are the upregulated proteins. **(B)** DE Pathways analysis of the groups 1-4 myotubes: skeletal muscle, splicing, intracellular organelles, cytoskeleton, signaling/cancer and enzymes, and nucleoside binding.

SUPPLEMENTARY FIGURE S4 Dysregulation of proteins of the groups 1-4 myotubes: Dysregulated proteins of the skeletal muscle are grouped based on their biological functions. Y ax logFC: below zero (downregulated proteins) and up zero (upregulated protein. X ax: name of the proteins. **(A)** constituent of the skeletal muscle (red), muscle contraction (green), adhesion, development, and differentiation(yellow), and actin stress (blue). **(B)** Dysregulated proteins in autophagy: lysosomal homeostasis (red), vesicular trafficking (green), mTOR pathway (purple), fusion membrane, binding protein (yellow), and clathrin endocytosis (blue). **(C)** Dysregulated proteins in the mitochondria: ATP generation: glycolysis, Krebs cycle and respiratory chain (red), mitochondrial ribosome and protein encoded by the mitochondrial DNA, mtDNA(green), Import proteins (purple), fission (yellow), calcium-binding (blue). **(D)** Cellular stress: DNA repair and transcription regulation (red), p53 activity regulation (green), protein degradation (purple), and disulfide bond cleavage (blue).

SUPPLEMENTARY FIGURE S5 MYF5 and MYOD1 protein levels in myoblasts and myotubes R155H/+ and WT p97 in Group 1 cells. In the Western Blot assay, we measured the MYF5 (28 KDa) and MYOD1 (35 KDa) protein levels in myoblast and myotubes of Group 1 that has three R155H/+ Clones (R155H/+ Clone 1, R155H/+ Clone 2, and R155H/+ Clone 3) and three WT (or Ctrl): Ctrl1, Ctrl 2, and Ctrl 3. These uncropped full-length blot images (Suppl. Fig. 5; A-B) were showed also as cropped blot images (Fig. 2; F-G).

## Abbreviations

INDEL: Insertion/Deletion
MHC: Myosin Heavy Chain
MYF5: Myogenic factor 5
MYOD1: Myogenic Differentiation 1
OCT-4: Octamer-binding transcription factor 4
PAX3: Paired box gene 3
PAX7: Paired box gene 7
SSEA-4: Stage-specific embryonic antigen-4
TRA-1-60: T cell receptor Alpha locus
VCP: Valosin Containing Protein
