# Supplementary figures and images for: Skeletal muscle cell protein dysregulation highlights the pathogenesis mechanism of myopathy-associated p97/VCP R155H mutations
Source: Front Neurol. 2023 Aug 3;14:1211635. doi: 10.3389/fneur.2023.1211635 (PMC10435852; doi:10.3389/fneur.2023.1211635)

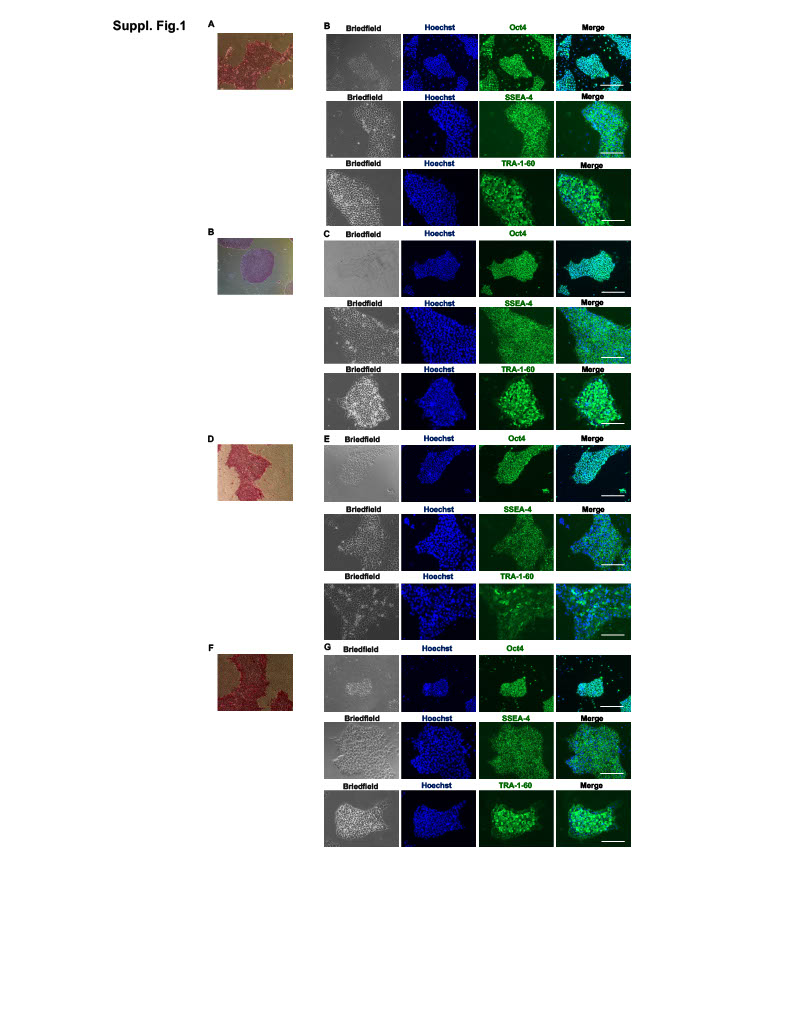

Supplement: SUPPLEMENTARY FIGURE S1 — Human iPSCs (hiPSCs) fibroblast-derived from patients harboring R155H mutation in the VCP/p97 gene and their screening selection of clones edited by the CRISPR/RNA method. iPSC diseased of groups 2-4 stained for (A) alkaline phosphatase. (B) pluripotency markers, Bright field, nuclei stained with Hoechst (Blue), Oct4; SSEA-4 and TRA-1-60 (green); Scale Barr 400nm. [file Image_1.JPEG]

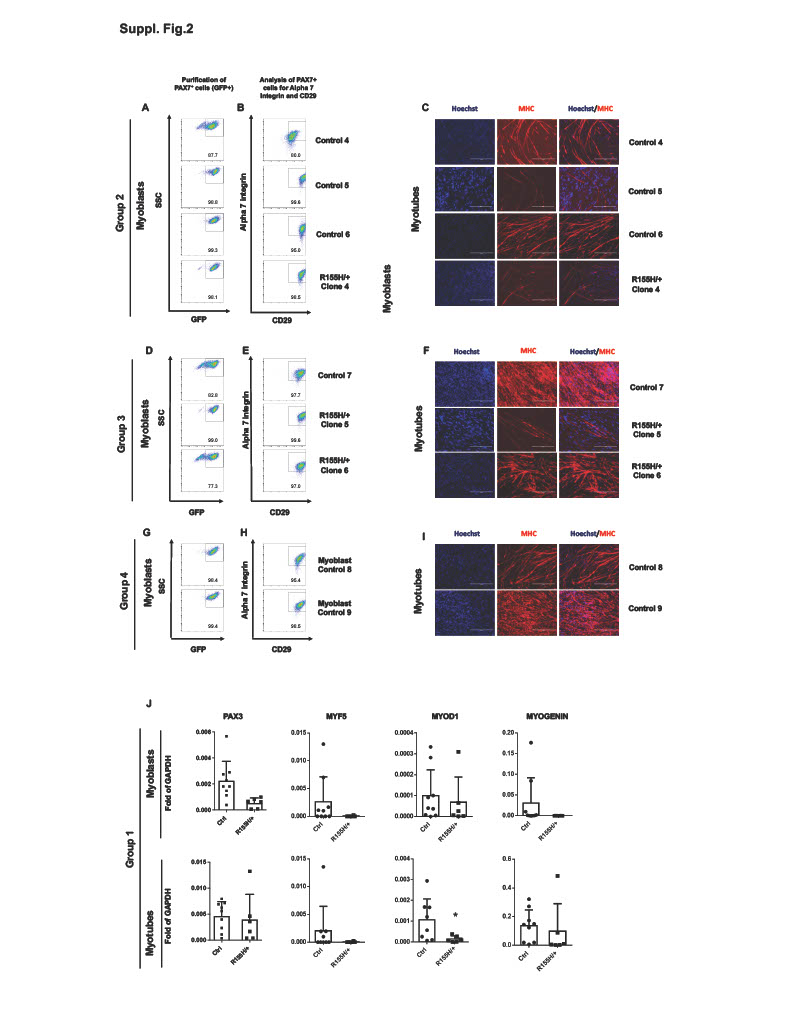

Supplement: SUPPLEMENTARY FIGURE S2 — Differentiation of R155H/+ and WT p97 hiPSC-Derived myogenic progenitors into muscle tissue following PAX7 induction. Group 2 (A-C), group 3 (D-F), and group 4 (G-I) myoblast were previously purified by FACS selection of only GFP positive (PAX7+) cells and then expanded Representative FACS profile of PAX7 induced proliferating myogenic progenitors (A, D, G). After 1 week of expansion, the myogenic precursors were stained for two early skeletal muscle markers, Alpha 7 integrin, and CD29. The percentage indicates cells staining positive for GFP, Alpha 7 Integrin, and CD29. SSC side scatters (B, E, H). Myogenic progenitors were differentiated into myotubes over 6-8 days, and immunofluorescence determined the myotube formation. Blue nuclei as stained with Hoechst. Red, Myosin Heavy Chain (MHC), myotubes; Scale Barr 400nm. [file Image_2.JPEG]

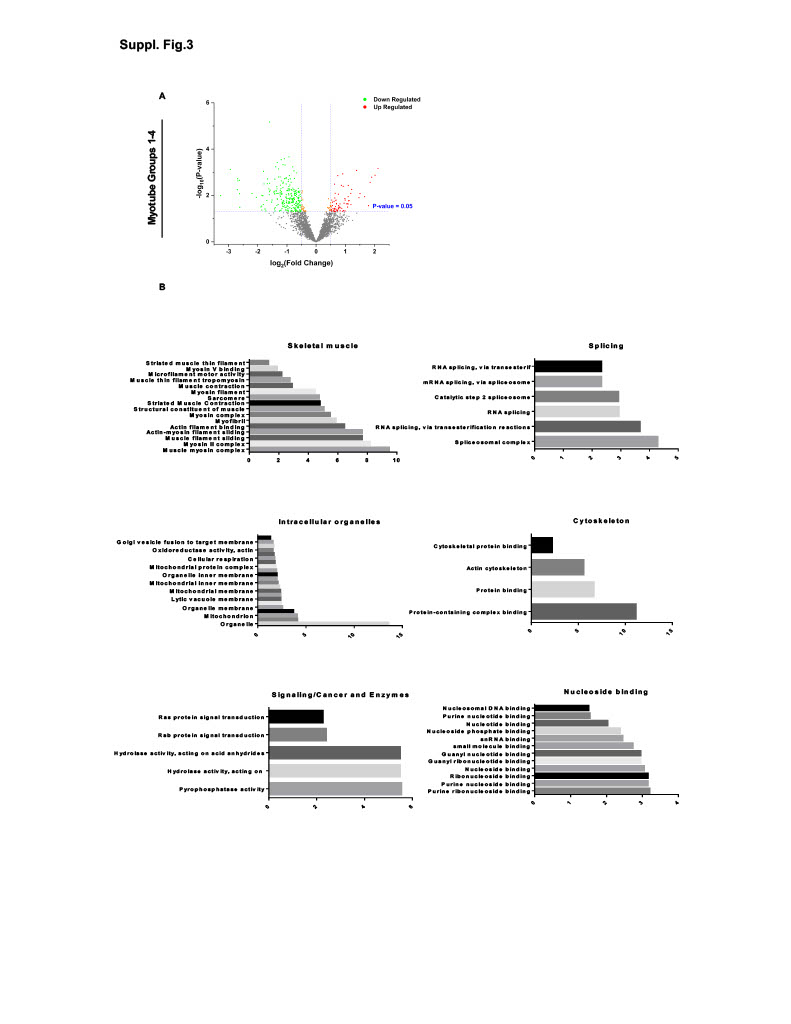

Supplement: SUPPLEMENTARY FIGURE S3 — Bioinformatics analysis of proteomic results. (A) Volcano plot analysis of statistically significant myotubes (P-value >0.05) in groups 1-4 myotubes. The P-value is represented in the Y ax, and the fold change in the X ax. The green dots are the down-regulated proteins, and the red dots are the upregulated proteins. (B) DE Pathways analysis of the groups 1-4 myotubes: skeletal muscle, splicing, intracellular organelles, cytoskeleton, signaling/cancer and enzymes, and nucleoside binding. [file Image_3.JPEG]

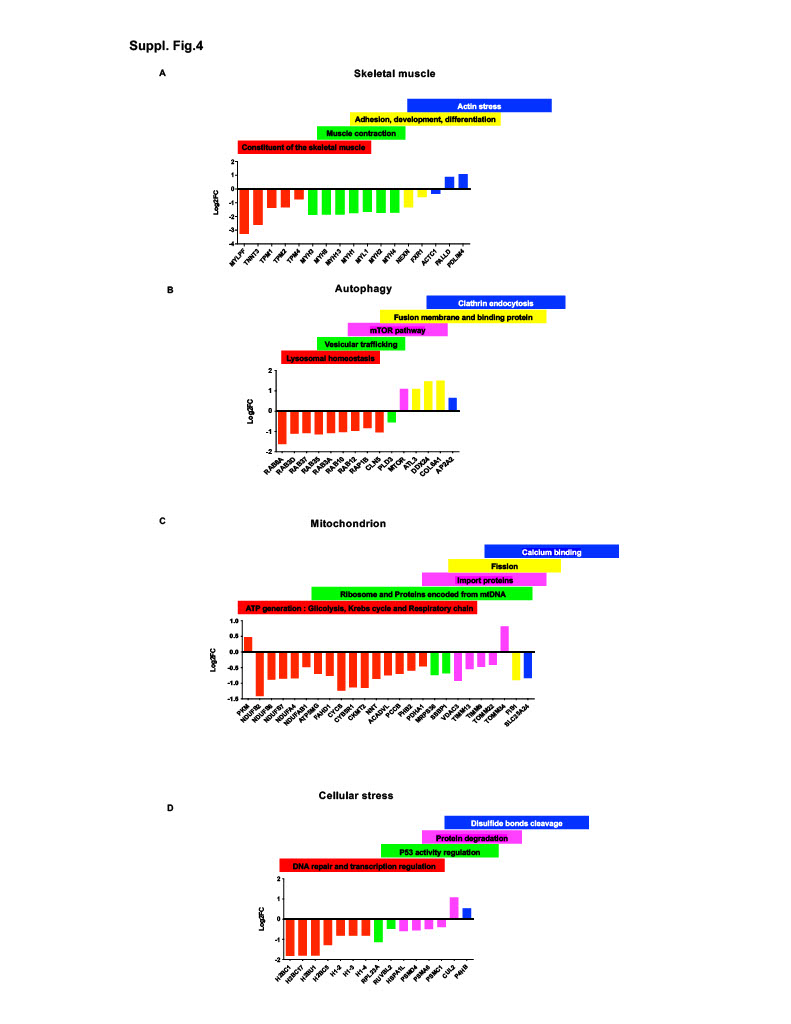

Supplement: SUPPLEMENTARY FIGURE S4 — Dysregulation of proteins of the groups 1-4 myotubes: Dysregulated proteins of the skeletal muscle are grouped based on their biological functions. Y ax logFC: below zero (downregulated proteins) and up zero (upregulated protein. X ax: name of the proteins. (A) constituent of the skeletal muscle (red), muscle contraction (green), adhesion, development, and differentiation(yellow), and actin stress (blue). (B) Dysregulated proteins in autophagy: lysosomal homeostasis (red), vesicular trafficking (green), mTOR pathway (purple), fusion membrane, binding protein (yellow), and clathrin endocytosis (blue). (C) Dysregulated proteins in the mitochondria: ATP generation: glycolysis, Krebs cycle and respiratory chain (red), mitochondrial ribosome and protein encoded by the mitochondrial DNA, mtDNA(green), Import proteins (purple), fission (yellow), calcium-binding (blue). (D) Cellular stress: DNA repair and transcription regulation (red), p53 activity regulation (green), protein degradation (purple), and disulfide bond cleavage (blue). [file Image_4.JPEG]

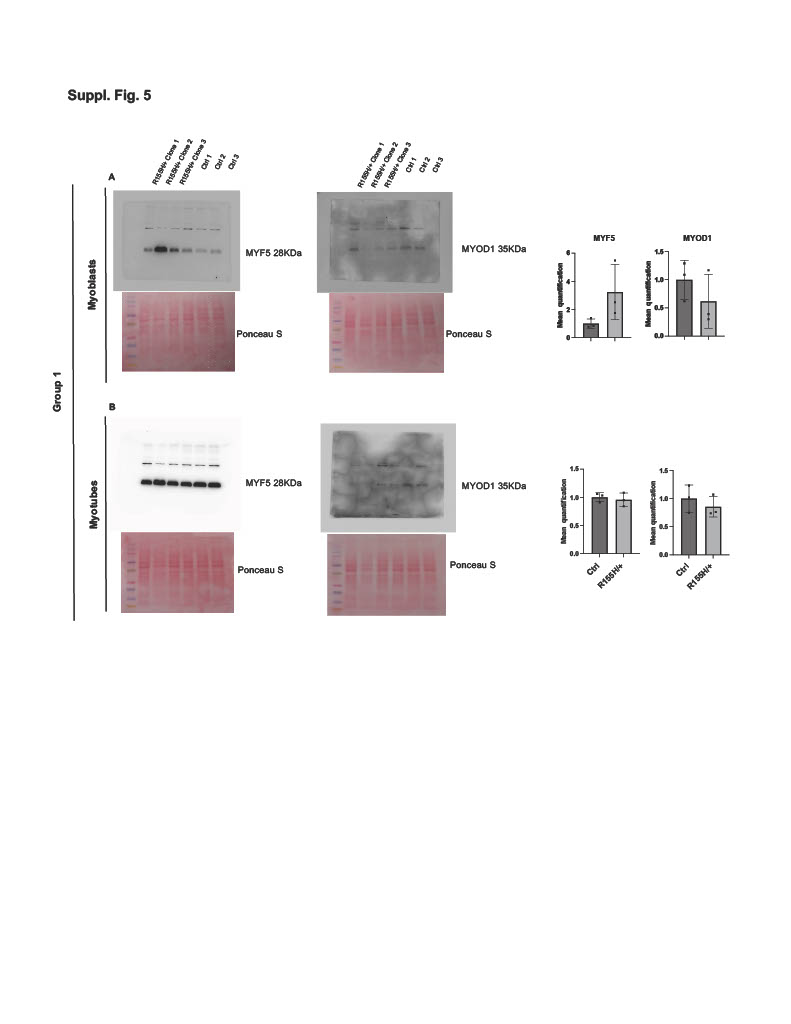

Supplement: SUPPLEMENTARY FIGURE S5 — MYF5 and MYOD1 protein levels in myoblasts and myotubes R155H/+ and WT p97 in Group 1 cells. In the Western Blot assay, we measured the MYF5 (28 KDa) and MYOD1 (35 KDa) protein levels in myoblast and myotubes of Group 1 that has three R155H/+ Clones (R155H/+ Clone 1, R155H/+ Clone 2, and R155H/+ Clone 3) and three WT (or Ctrl): Ctrl1, Ctrl 2, and Ctrl 3. These uncropped full-length blot images (Suppl. Fig. 5; A-B) were showed also as cropped blot images (Fig. 2; F-G). [file Image_5.JPEG]
